# Impact of Ischemic Intra-Conditioning on Power Output and Bar Velocity of the Upper Limbs

**DOI:** 10.3389/fphys.2021.626915

**Published:** 2021-02-25

**Authors:** Michal Wilk, Michal Krzysztofik, Jakub Jarosz, Pawel Krol, Katarzyna Leznicka, Adam Zajac, Petr Stastny, Gregory C. Bogdanis

**Affiliations:** ^1^Institute of Sport Sciences, The Jerzy Kukuczka Academy of Physical Education, Katowice, Poland; ^2^College of Medical Sciences, Institute of Physical Culture Studies, University of Rzeszów, Rzeszów, Poland; ^3^Faculty of Physical Education, Gdansk University of Physical Education and Sport, Gdańsk, Poland; ^4^Faculty of Physical Education and Sport, Department of Sport Games, Charles University, Prague, Czechia; ^5^School of Physical Education and Sport Science, National and Kapodistrian University of Athens, Athens, Greece

**Keywords:** blood flow restriction, occlusion, resistance exercise, bench press, sport performance

## Abstract

This study evaluated the effects of ischemic conditioning on power output and bar velocity in the bench press exercise. Ten healthy males (age: 25 ± 2 years; body mass: 92 ± 8 kg; bench press one repetition maximum −1RM: 145 ± 13 kg), took part in two experimental sessions (with and without ischemia), 1 week apart in random and counterbalanced order. In the ischemic condition, cuffs placed around the upper part of the arms were inflated to 80% of arterial occlusion pressure before each set, while in the control condition there was no blood flow restriction. The exercise protocol included 5 sets of three repetitions each, against a resistance equal to 60% 1RM, with 5 min recovery intervals between sets. There was a main effect of condition for mean power output (MP) and mean bar velocity (MV) (*p* = 0.01), with overall MP being higher in ischemia than in control by 5.6 ± 4.1% (mean ± 90% compatibility limits), a standardized effect size (ES) of 0.51. Overall MV was also higher by 5.5 ± 4.0%, ES = 0.63. Peak power output (PP) and peak bar velocity (PV) were similar in set 1 of the control and ischemia condition (1039 ± 105 vs. 1054 ± 82 W; 684 ± 74 vs. 696 ± 53 W; 1.09 ± 0.07 vs. 1.12 ± 0.09 m/s; 0.81 ± 0.05 vs. 0.82 ± 0.05 m/s, *p* = 0.67 to 0.99, mean ± standard deviation). However, from set 3 onward (*p* = 0.03 to 0.001), PP and PV were higher in ischemia compared with control, with the highest difference observed in set 5 (10.9 ± 5.9%, ES = 0.73 for PP and 8.6 ± 4.6%; ES = 0.89 for PV). These results indicate that ischemia used before each set of the bench press exercise increases power output and bar velocity and this may be used as performance-enhancing stimulus during explosive resistance training.

## Introduction

Ischemia, also referred to as blood flow restriction and occlusion, can be used in any form of physical activity and much attention has been devoted to the use of ischemia during resistance training (Wilk et al., 2018). Partial or total ischemia of working muscles during resistance exercise, has been used as a complementary training modality, aiming to further increase muscle mass, enhance strength and acute power output and maximal load lifted (Wilk et al., 2018, 2020e,g; Wernbom et al., 2019, 2020; Gepfert et al., 2020). There are several options for using ischemia as part of resistance training: constant (used during exercise and during the rest interval); intermittent (used only during exercise) (Wilk et al., 2020a), and ischemic preconditioning (used only before exercise) (Schroeder et al., 1996).

Resistance training with ischemia leads to increased metabolic stress (Pearson and Hussain, 2015; Teixeira et al., 2018), cell swelling (Loenneke et al., 2012), enhanced intramuscular signaling (Manini et al., 2011; Laurentino et al., 2012), increased recruitment of fast twitch muscle fibers (Takarada et al., 2000; Manimmanakorn et al., 2013), and enhanced responses of the endocrine system (Takano et al., 2005; Shimizu et al., 2016). Furthermore, the hyperemia experienced after ischemia may play a significant role in nitric oxide production (Singh et al., 2017), increased phosphocreatine resynthesis, altered oxy-deoxyhemoglobin kinetics (Bailey et al., 2012), and increased oxygen uptake (Andreas et al., 2011). The ischemic preconditioning stimulate the release of nitric oxide (Kimura et al., 2007; Li et al., 2012), as well as the activation of the adenosine receptors (Liu et al., 1991; Schroeder et al., 1996), causing vasodilation after blood reperfusion (Kimura et al., 2007; Li et al., 2012), increasing O_2_ extraction by the muscle (Paradis-Deschênes et al., 2016; Tanaka et al., 2018), and opening of adenosine triphosphate (ATP)-dependent potassium (K^+^) channels which increase the energy stores after ischemia (Lawson and Downey, 1993; Pang et al., 1995).

Despite that, much attention has been focused on ischemia used during resistance training, only few previous studies have compared the acute effects (Luebbers et al., 2014; Teixeira et al., 2018; Rawska et al., 2019; Gepfert et al., 2020; Wilk et al., 2020b,g), and only two studies have examined its effects on subsequent power output performance in the upper limbs (Wilk et al., 2020b,g). Wilk et al. (2020b) showed that intermittent (used only during exercise), ischemia increases bar velocity and power output during the bench press exercise at 70% one-repetition maximum (1RM). However, such potential benefits were obtained only under high pressure and wide cuff (10 cm), while no benefits were observed under a narrow cuff (4 cm) (Wilk et al., 2020g). Further Wilk et al. (2020b) showed that ischemia (continuous as well as intermittent) with a narrow cuff can increase bar velocity during the bench press exercise at loads of 20 to 50%1RM. Although ischemia used during resistance exercise increases acute power output and velocity of movement (Gepfert et al., 2020; Wilk et al., 2020b,g), there is no scientific data assessing the effect of ischemia used only during the rest interval between successive sets of resistance exercise. The use of ischemia during the rest interval between sets can produce similar effects to those induced by ischemic preconditioning and improve exercise performance (Lawson and Downey, 1993; Pang et al., 1995; Incognito et al., 2016; Marocolo et al., 2015, 2016, 2017, 2018). However, the effectiveness of ischemic preconditioning interventions in competitive sports remains unclear. Indeed, some studies report significant exercise performance benefits (i.e., time trial performance, maximal oxygen uptake (VO_2__max_), power output), whereas others demonstrate no effect (Incognito et al., 2016; Marocolo et al., 2016).

Given the ischemia used during the rest interval between successive sets can induce and maintain similar effects and physiological responses to those induced by ischemic preconditioning it would be interesting to examine its effects on bar velocity and power output during resistance exercise. Since the bench press is a basic resistance exercise for developing upper body strength and power (Dunnick et al., 2015; Stastny et al., 2017; Wilk et al., 2019a, 2020d), the present study aimed to evaluate the effects of ischemia used during the rest interval between successive sets of the exercise on bar velocity and power output during the bench press exercise. It was hypothesized that ischemic intra-conditioning would increase bar velocity and power output during the bench press exercise.

## Materials and Methods

The experiment was performed following a randomized crossover design, where each subject performed two different testing protocols in random and counterbalanced order, 1 week apart: with ischemia used only during the rest interval between sets and control condition without ischemia. Before the main tests, two familiarization sessions were performed. One week before the first session, a maximal bench press test (1 repetition maximum-1RM) was performed. During each experimental session, the participants performed 5 sets of three repetitions each, with a load of 60%1RM, and a 5 min rest interval between each set. Each repetition was performed with maximal tempo in the eccentric and concentric phase of the bench press movement (Wilk et al., 2020a,c). The following variables were measured using a linear position transducer: peak bar velocity, mean bar velocity, peak power output, and mean power output. All testing sessions were performed in the Strength and Power Laboratory at the Academy of Physical Education in Katowice, Poland.

### Subjects

Ten healthy resistance trained men volunteered for the study after completing an informed consent form (age = 25 ± 2 years; body mass = 92 ± 8 kg; bench press 1RM = 145 ± 13 kg; bench press 1RM/body mass = 1.6 ± 0.1). The main inclusion criteria were: a bench press personal best of at least 120% body mass (Wilk et al., 2019b; Maszczyk et al., 2020), and that the subject was free from musculoskeletal injuries for at least 6 months prior to the study. The participants were instructed to maintain their normal dietary habits over the course of the study and not to use any supplements or stimulants for the duration of the experiment. They were informed about the benefits and potential risks of the study before providing their written informed consent for participation and were allowed to withdraw from the study at any time. The study protocol was approved by the Bioethics Committee for Scientific Research, at the Academy of Physical Education in Katowice, Poland (02/2019), and all procedures were in accordance with the ethical standards of the Declaration of Helsinki, 1983.

### Procedures

#### Familiarization Session and the 1RM Strength Test

Two weeks before the main experiment, the subjects performed two familiarization sessions. During the familiarization sessions, each subject performed 5 sets of three repetitions of the bench press exercise with ischemia used only during the rest interval with a load of 60% of their estimated 1RM. The familiarization sessions were performed in order to minimize possible learning effects during the main tests.

One week before the main experiment the 1RM bench press test was performed. On arrival, body mass was measured and then the subjects cycled on a cycle-ergometer for 5 min, followed by a general upper body warm-up as described elsewhere (Wilk et al., 2019a). Then, the subjects performed 15, 10, and 5 bench press repetitions using 20, 40, and 60% of their estimated 1RM. The first testing load was set to an estimated 80%1RM, and was increased by 2.5 to 10 kg for each subsequent attempt. This process was repeated until failure. The rest interval between successful trials was 5 min. Hand placement on the barbell was set at 150% of the individual bi-acromial distance, and this was used for all main trials (Wilk et al., 2019a).

#### Experimental Sessions

In a randomized and counterbalanced order, the participants performed the bench press exercise under two different testing conditions: ischemia condition (ischemia used only during the rest interval between sets); control condition (without ischemia). During each testing protocol the subject performed 5 sets of the bench press at a load of 60%1RM with a 5 min rest interval between sets. During each set the subjects performed three repetitions, with a maximal tempo of movement (Wilk et al., 2020a,c). A linear position transducer system (Tendo Power Analyzer, Tendo Sport Machines, Trenčín, Slovakia) was used for the evaluation of bar velocity. Measurements were made independently for each repetition and automatically converted into values of concentric peak and mean bar velocity (PV and MV, respectively), as well as concentric peak (PP) and mean power output (MP). The peak values were obtained from the best repetition performed in a particular set. The mean values were obtained from the mean of three repetitions performed in particular sets. All subjects completed the described testing protocol that was carefully replicated in subsequent experimental sessions.

#### Ischemia Procedure

During the ischemia condition, the participants wore pressure cuffs in close proximity to the axillary’s fossa of both arms. For this experiment Smart Cuffs were applied (Smart Tools Plus LLC, Strongsville, OH, United States). The individual cuff pressure was determined immediately before the ischemic session. To determine the individual pressure, after completion of the general warm-up and 5 min rest interval, the value of full arterial occlusion pressure was determined. The measurement was conducted twice on each limb and the obtained differences were within 20 mmHg, with the average value used to set the cuff pressure for the exercise protocol (Wilk et al., 2020e,g). The cuff pressure for the bench press exercise was set to ∼80% of full arterial occlusion pressure (116 ± 7 mmHg). The level of vascular restriction was monitored using a handheld Edan SD3 Doppler with an OLED screen and a 2 mHz probe made by Edan Instruments (Shenzhen, China). For the ischemia protocol, the cuff was applied immediately after the termination of the set and released upon the start of the next one. For the ischemia protocol the cuff was also applied 5 min before the first set.

#### Statistical Analysis

All statistical analyses were performed using Statistica 9.1. Results are presented as means with standard deviations. The Shapiro-Wilk, Levene and Mauchly’s tests were used in order to verify the normality, homogeneity and sphericity of the sample data variances, respectively. Data were log-transformed using 100 × ln(y), where y is the dependent variable (Hopkins et al., 2009). Differences between the ischemia and control conditions were examined using repeated measures two-way ANOVA of the log-transformed data (2 conditions × 5 sets). The statistical significance was set at *p* < 0.05. *Post hoc* comparisons using the Tukey’s test were conducted to locate the differences between mean values when a main effect or an interaction was found. To compare the changes in performance during the two conditions, changes of performance at corresponding sets were calculated from the log-transformed data, along with the appropriate 90% compatibility limits. To express these differences in percent values, data were back-transformed using the following formula: 100 × exp(×/100)−100, where × are the differences from set 1 (Hopkins et al., 2009). Pairwise comparisons between performance in corresponding sets were done by calculating effect sizes (ES) determined by Hedge’s g, using the standard deviation of the control condition. Hedge’s g was characterized as large (*g* > 1.2); moderate (g between 0.6 and 1.2) and small (g between 0.2 and 0.59) (Hopkins et al., 2009).

## Results

**FIGURE 1:**
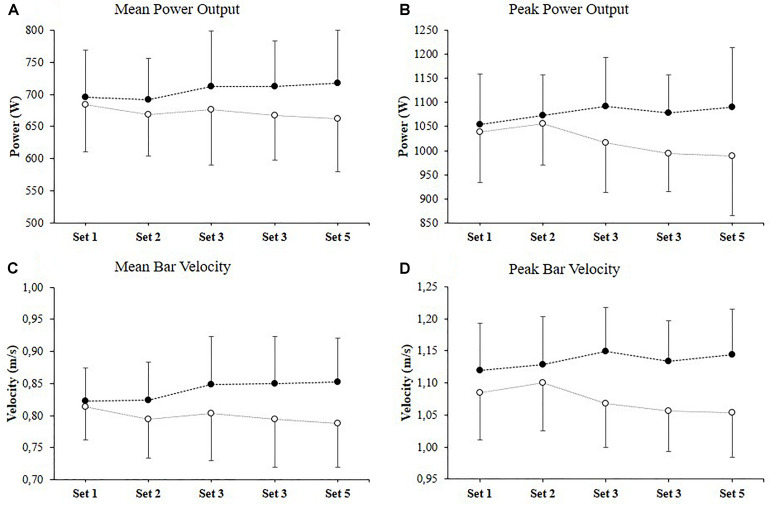
Power output and bar velocity during five successive sets of the bench press exercise in the ischemia (filled symbols) and control conditions (open symbols). Values are expressed as mean ± standard deviation. **(A)** Mean power output; **(B)** Peak power output; **(C)** Mean bar velocity; **(D)** Peak bar velocity.

The two-way repeated measures ANOVA showed statistically significant interaction for PP (conditions × sets; *p* = 0.03) and for PV (conditions × sets; *p* = 0.04). The *post hoc* analysis revealed that PP and PV were similar in set 1 of the control and ischemia condition (1039 ± 105 vs. 1054 ± 82 W; 684 ± 74 vs. 696 ± 53 W; 1.09 ± 0.07 vs. 1.12 ± 0.09 m/s; 0.81 ± 0.05 vs. 0.82 ± 0.05 m/s, *p* = 0.67 to 0.99). However, PP was significantly higher in ischemia condition when compared to control in set 3 (*p* = 0.05), set 4 (*p* = 0.03) and set 5 (*p* = 0.003; Table 1; Figure 1). Similarly, PV was significantly higher in ischemia condition compared with control in set 3 (*p* = 0.003), set 4 (*p* = 0.004) and set 5 (*p* < 0.001; Table 2; Figure 1). The highest difference between ischemia and control was observed in set 5 for both PP (10.9 ± 5.9%, [mean ± 90%CI], ES = 0.73) and PV (8.6 ± 4.6%; ES = 0.89).

**TABLE 1:** Mean differences between the control and the ischemia conditions for peak and mean power output in corresponding sets.

	Difference in peak power (mean ± 90%CL)	Hedge’s g	Difference in mean power (mean ± 90%CL)	Hedge’s g
Set 1	1.9 ± 3.5%	0.16	2.2 ± 3.1%	0.20
Set 2	1.7 ± 4.0%	0.17	3.9 ± 3.9%	0.39
Set 3	7.9 ± 4.7%	0.74	6.1 ± 4.1%	0.52
Set 4	8.5 ± 4.6%	0.80	7.1 ± 4.6%	0.62
Set 5	10.9 ± 5.9%	0.73	8.8 ± 4.6%	0.67

*The effect size for pairwise comparisons of corresponding sets was calculated using Hedge’s g. 90%CL: mean ± 90% compatibility limits.*

**TABLE 2:** Mean differences between the control and the ischemia conditions for peak and mean bar velocity output in corresponding sets.

	Difference in peak bar velocity (mean ± 90%CL)	Hedge’s g	Difference in mean bar velocity (mean ± 90%CL)	Hedge’s g
Set 1	3.1 ± 2.5%	0.39	1.2 ± 2.4%	0.17
Set 2	2.6 ± 3.1%	0.30	4.0 ± 3.9%	0.48
Set 3	7.5 ± 3.7%	0.82	6.1 ± 4.0%	0.66
Set 4	7.3 ± 3.9%	0.84	7.4 ± 4.5%	0.70
Set 5	8.6 ± 4.6%	0.89	8.6 ± 4.5%	0.91

*The effect size for pairwise comparisons of corresponding sets was calculated using Hedge’s g. 90%CL: mean ± 90% compatibility limits.*

There was no significant condition × set interaction for mean power output (*p* = 0.19) and mean bar velocity (*p* = 0.12). However, there was a main effect of condition for MP and MV (*p* = 0.01), with overall MP and MV being higher in ischemia than in control (by 5.6 ± 4.1%; ES = 0.51 and by 5.5 ± 4.0%; ES = 0.63, respectively).

Tables 1, 2 show the percent between the control and the ischemia conditions for peak and mean power output in corresponding sets. In the control condition performance was decreased from set 3 onward, while in the ischemia condition an increase in performance was observed for all variables. Comparisons of percent changes in corresponding tests showed moderate to large effect sizes from set 3 onward (Hegde’s g = 0.52 to 0.91).

## Discussion

The main finding of the study was that ischemic intra-conditioning significantly increased power output and bar velocity during the bench press exercise at 60%1RM. Our study showed that both mean and peak power output and bar velocity were significantly higher during 5 sets of the bench press exercise for the ischemia condition compared to the control one. Further detailed analysis independently for each set showed significantly higher peak power output and peak bar velocity from set 3 to set 5 for the ischemia condition compared to control. Therefore, the results of this study indicate that ischemia used during the rest interval between successive sets can effectively improve upper body strength performance.

To the best of the author’s knowledge, currently there are no available analysis related to the impact of ischemia used during the rest interval between successive sets of resistance exercise on power output and bar velocity changes, which limits the possibility of comparing our results with other studies. Nevertheless, significant knowledge and training clues can be derived from the current data. The increase in performance observed for ischemia condition can be related with a similar physiological factor to those induced by ischemic preconditioning, such as the release of nitric oxide (Kimura et al., 2007; Li et al., 2012), activation of the adenosine receptors (Liu et al., 1991; Schroeder et al., 1996), increasing the O_2_ extraction by the muscle (Paradis-Deschênes et al., 2016; Tanaka et al., 2018), and opening of adenosine triphosphate (ATP)-dependent potassium (K^+^) channels which increase the energy stores after ischemia (Lawson and Downey, 1993; Pang et al., 1995). Another factor that could increase the power output and bar velocity when the ischemia was used may be related with the bidirectional brain-body integration mechanism activated after ischemia which may promote physiological responses through mechanical sensory receptors (Taylor et al., 2010; Cromwell and Panksepp, 2011; de Souza et al., 2019), thus increasing the effectiveness of resistance exercise (de Souza et al., 2019).

It is interesting to note that the increase of performance for ischemia condition was observed particularly in sets 3 to 5 (Figure 1), which suggests that ischemia allows the athletes to maintain a certain amount of force even in the presence of biochemical changes within the working muscle that lead to fatigue. Such an effect was also observed in the study of Wilk et al. (2020b), which showed that both intermittent (used only during exercise) and continuous (used during exercise and during the rest interval) ischemia significantly increased peak bar velocity during the bench press exercise for lighter loads (20 to 50%1RM), while not for higher ones (60–90%1RM). Despite, the lack of an increase in bar velocity at higher loads during ischemia condition in study Wilk et al. (2020b), the level of performance was maintained while, our research showed not only a maintenance of performance for the ischemia condition, but an increase in power output and bar velocity compared to control condition. However, contrary to Wilk et al. (2020b) in presented study the ischemia was used only during the rest intervals and not during the exercise, which suggests that different ischemia protocols may be an important factor in affecting on acute responses. Furthermore, similar as with ischemia used during exercise, the ischemia used only during the break intervals, there may be an interaction between the resistive load, the width of the cuff, and the pressure in the cuff, which determines the size of the acute changes (Loenneke et al., 2012; Rossow et al., 2012).

The increased performance during successive sets of the bench press for ischemia condition can also be attributed to the more efficient use of post-activation performance enhancement. The post-activation performance enhancement is a unique phenomenon which allows short term improvement of voluntary force and power output production due to a prior muscle stimulation (Krzysztofik and Wilk, 2020; Krzysztofik et al., 2020a,b; Wilk et al., 2020d). Some studies have shown a beneficial effect of previous muscle activation on performance during throwing, jumping, and hitting movements (Proske and Morgan, 2001; Golas et al., 2017), as well as a stimulating effect of muscle activation on power output generated in consecutive sets of a single resistance exercise (Wilk et al., 2020d,f). Therefore the increase in power output and bar velocity in successive for ischemia condition can be related to the more effective use of the post-activation performance enhancement effect. This is confirmed by the difference in power output and bar velocity in successive sets between ischemia and control conditions, which increased from trivial and small ES in the first set (0.16–0.39) to moderate and high ES in the fifth set (0.66–0.91). Therefore, the use of ischemic intra-conditioning not only maintains the level of performance during several sets of a resistance exercise but increases power output and bar velocity in the following sets compared to the control condition. Such a performance increase in the following sets suggests that repeated application of ischemia during a training session may enhance its positive effect on power output and bar velocity (Figure 1). However, factor that can have a significant impact on the level of the post-activation performance enhancement includes subject characteristics, especially the level of strength (Seitz and Haff, 2016; Krzysztofik et al., 2020a,b). In presented study the participants were a homogeneous group with a relatively high level of strength and small differences between the participants (ratio of 1RM bench press to body weight = 1.6 ± 0.1), which could have influenced the obtained results.

Previous studies have shown that the ratio of exercise duration to rest is the major factor which induces post-activation performance enhancement, also in case of successive sets of a resistance exercise (Chaouachi et al., 2011; Gossen and Sale, 2000; Wilk et al., 2020d). Therefore, the duration of effort, and the ratio of effort to rest can be a significant factor which induces positive effects on power output following ischemia. The presented research procedure involved a low number of repetitions (3 reps) lasting approximately 3–5 s. Such brief efforts are fueled mainly by phosphocreatine and anaerobic glycolysis (Bird et al., 2005), which are largely restored during the rest interval (Bogdanis et al., 1998; Dawson et al., 2007), which may be of particular importance in the efficient use of ischemic intra-conditioning. Performing repetitions to exhaustion in each set or shortening the rest internal would be expected to cause significant reductions in exercise capacity (Wernbom et al., 2009), limiting the benefits of using ischemia.

Although the results of the present study showed that ischemic intra-conditioning during resistance exercise may be used to enhance performance during upper body resistance exercises, there are certain limitations of the study which should be addressed. Although the results showed that ischemia increases power output and bar velocity, during the bench press exercise at 60%1RM, the causes of these changes could not be determined and explained due to the lack of physiological and biomechanical evaluations, which could provide possible explanations. Furthermore, the results of this study may not translate to other types of exercises, different loads or different ischemic procedures, as well as different cuff pressure and thus further research is required.

## Practical Implications

The results of this study have significant implications for resistance training. The present study showed that ischemia used between successive sets of the bench press exercise can be effectively used to increase power output and bar velocity during resistance exercises of the upper body. Therefore, the use of ischemia between sets of resistance exercise seems to be particularly valuable for sport disciplines, that require repeated upper-limb explosive movements. Moreover, it should be indicated that ischemic intra-conditioning during resistance training could induce additional physiological responses (not evaluated in the present study). Therefore, the use of ischemia during resistance exercise may increase physiological responses which can be a significant factor determining the level of post-exercise adaptive changes. The primary disadvantage of this method relates to the weakening of the musculature in the area of direct application of the cuff (Kacin and Strazar, 2011; Ellefsen et al., 2015). Therefore, coaches and athletes should introduce ischemia protocols with caution and gradually progress with the training loads over time, to ensure that protective adaptations (i.e., a repeated bout effect) can take place in order to minimize the risk of excessive muscle stress and damage (Clark and Manini, 2017; Wernbom et al., 2019).

## Conclusion

The results of the present study indicated that the ischemic intra-conditioning used between sets during resistance exercise increases power output and bar velocity and thus can be useful for improving explosive performance in sports training. Such improvements were observed for both mean and peak value of power output and bar velocity in the ischemia condition. Furthermore, increasing differences between the ischemia and control conditions observed in successive sets suggests that ischemia used during the rest interval may delay symptoms of fatigue. Therefore, the use of ischemia, may maintain the level of performance, and also increase power output and bar velocity when compared to baseline and control conditions.

## Data Availability Statement

The raw data supporting the conclusions of this article will be made available by the authors, without undue reservation.

## Ethics Statement

The studies involving human participants were reviewed and approved by the Bioethics Committee for Scientific Research, at the Academy of Physical Education in Katowice, Poland (02/2019). The patients/participants provided their written informed consent to participate in this study.

## Author Contributions

MW, MK, and JJ: study conception and design. MW, PK, KL, and JJ: acquisition of data. MW, MK, PS, and GB: analysis and interpretation of data. MW: drafting of the manuscript. MW, MK, AZ, and GB: critical revision. All authors contributed to the article and approved the submitted version.

## Conflict of Interest

The authors declare that the research was conducted in the absence of any commercial or financial relationships that could be construed as a potential conflict of interest.
